# PEGylated Nanoliposomes Encapsulated with Anticancer Drugs for Breast and Prostate Cancer Therapy: An Update

**DOI:** 10.3390/pharmaceutics17020190

**Published:** 2025-02-04

**Authors:** Sijongesonke Peter, Vuyolwethu Khwaza, Sibusiso Alven, Tobeka Naki, Blessing Atim Aderibigbe

**Affiliations:** Department of Chemistry, University of Fort Hare, Alice 5700, South Africa; 201414787@ufh.ac.za (S.P.); vkhwaza@ufh.ac.za (V.K.); 201214199@ufh.ac.za (S.A.)

**Keywords:** chemotherapeutics, PEG, toxicity, nanoliposomes, anticancer, drug resistance, bioavailability, breast cancer, prostate cancer

## Abstract

There are different types of cancer treatments, including surgery, radiotherapy, and chemotherapy. However, the complexity of cancer has resulted in treatment challenges to medicinal scientists and a socio-economic burden to the public health system globally. The pharmacological limitations associated with the current conventional anticancer drugs include lack of specificity, poor bioavailability, toxicity, drug resistance, and poor delivery mechanisms, which make cancer treatment challenging. Thus, the number of cancer cases is escalating rapidly, especially breast and prostate cancer in women and men, respectively. The application of nanoformulations is gaining momentum for treating different cancer types. However, they also exhibit challenges that must be addressed for effective cancer treatment. Nanoliposomes are nanoformulations that are widely explored for cancer treatment with interesting therapeutic outcomes. They have been functionalized with PEG to further improve their therapeutic outcomes. Hence, this review provides an update on PEGylated nanoliposomes loaded with anticancer drugs for the treatment of breast and prostate cancer, focusing on pre-clinical studies published in the last decade (2015 to 2024) to reflect the recent advancements made in the design of PEGylation nanoliposomes. Highlights of the clinically and commercially available PEGylation nanoliposomes are also presented in this review.

## 1. Introduction

The rise in global cancer cases is a significant concern for medicinal scientists and the public health system. Cancer is treated via surgery, radiotherapy, and chemotherapy, and due to some limitations, chemotherapy is the most popular and commonly used strategy nowadays [1]. However, multidrug resistance is a challenge with most chemotherapeutic agents. Hence, more than 19 million cases and 10 million deaths (cancer-related) were reported in 2020 globally [1,2]. Among cancer-related cases, breast and prostate cancer are the most prevalent cancer types in women and men, respectively. In 2023, there were about 297,790 cases of breast cancer and 288,300 cases of prostate cancer in the US alone, with estimated deaths of 43,170 and 34,700, respectively [3]. Chemotherapy such as anticancer drugs is the primary method for treating these cancer types. Despite their effectiveness, anticancer drugs face several limitations that hinder their use in cancer treatment.

**Figure 1:**
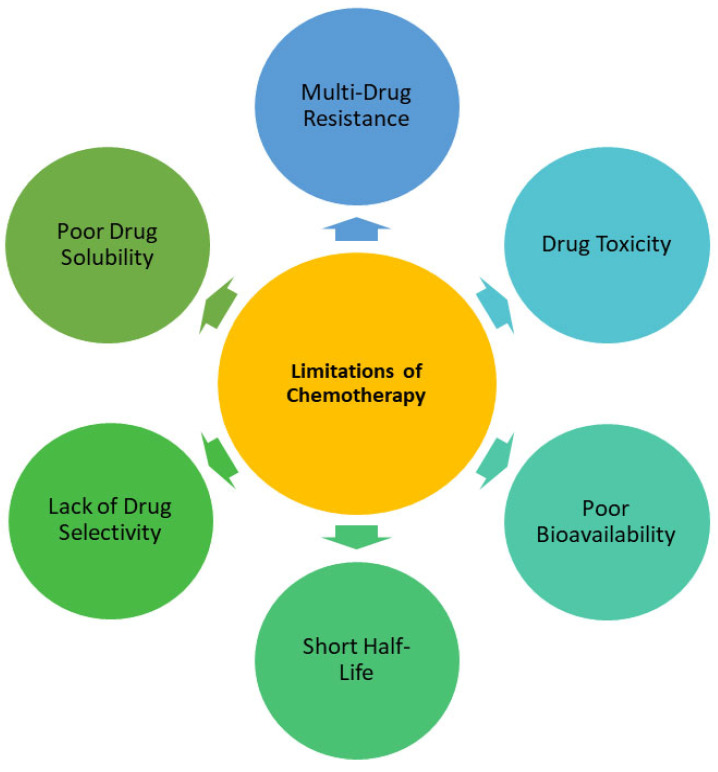
Limitations of Chemotherapy.

Among the limitations of chemotherapy are the lack of selectivity, toxicity, insufficient drug delivery to the desired site, poor drug solubility, and low bioavailability (Figure 1). In essence, poor drug delivery to the targeted tumor is one of the major drawbacks hampering the positive outcomes of chemotherapy [1,2,4,5,6]. Therefore, a better alternative to treating cancer is a pressing need. Nano-sized drug delivery systems have been widely documented as promising therapeutic tools for combating cancer. Nanocarriers are drug delivery systems with diameters between 1 and 100 nm. Nanocarriers studied in cancer research include nanoliposomes, micelles, dendrimers, nanoparticles, polymer-drug conjugates, and nanocapsules [7]. Nanoliposomes have been explored as effectual systems for drug delivery of anticancer drugs for breast and prostate cancer targeting. Nanoliposomes offer several notable advantages that have captured the interest of biomedical researchers. These include precise drug delivery, a reduction in the toxic effects of the loaded drugs on healthy human cells, and the ability to control and sustain the release of drugs over time. Additionally, nanoliposomes demonstrate excellent biocompatibility and enhance the overall therapeutic effectiveness of treatments. The other interesting advantages of nanoliposomes include prolonged drug clearance and circulation half-life [8,9].

**Figure 2:**
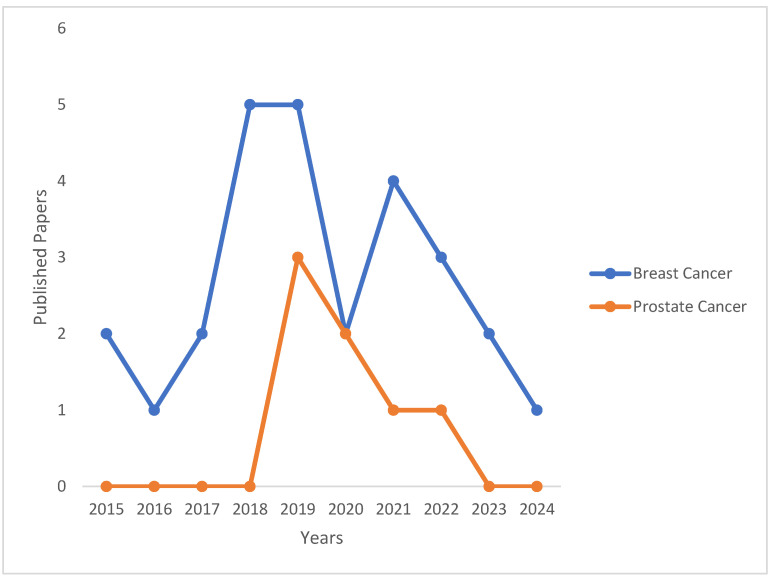
Research trends in the PEGylation of nanoliposomes for breast and prostate cancer by the number of publications per year, 2015–2024. (Source: Scopus. Keywords: nanoliposomes for breast cancer, nanoliposomes for breast cancer therapy. The keywords were used to select the relevant articles, and the search was limited to 2015 to 2024).

Nanoliposomes can be functionalized with polymers (i.e., natural or synthetic polymers) to improve their therapeutic outcomes further. The natural polymers that can be utilized for the functionalization of nanoliposomes are gelatin, cellulose, chitosan, hyaluronic acid, etc. [10,11,12]. Synthetic polymers include (poly-ethylene glycol) (PEG), PLGA, Poly(2-ethyl-2-oxazoline) (PEtOx), poly (ε-caprolactone), etc. [13,14]. PEG has been explored extensively for synthesizing liposomes to deliver bioactive agents for cancer targeting. The technology of PEGylation (PEGylation) was first introduced in the late 1960s by Frank Davis to avoid immunogenic reactions and enzyme attacks of a recombinant protein drug [15]. Presently, PEG is approved by the US Food and Drug Administration (FDA) as a carrier in numerous formulations, including pharmaceuticals. Incorporating PEGylated nanoliposomes composed of a longer PEG chain reduces pH responsiveness, though the cell uptake toward the high PEG molecules can be low [16]. This review manuscript discusses PEGylated nanoliposomes, which are loaded with anticancer drugs, for treating breast and prostate cancer. The number of publications per year on PEGylated nanoliposomes for breast and prostate cancer therapy is shown in the line graph below (Figure 2).

## 2. PEGylation Technology in Nanoliposomal Formulations

**Figure 3:**
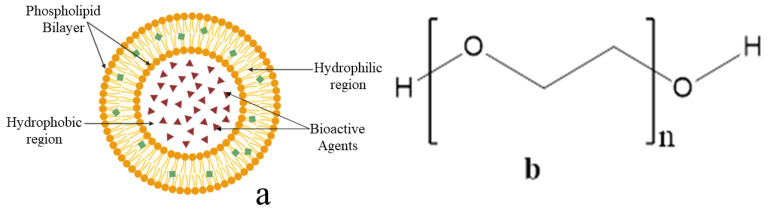
(**a**) Nanoliposomes encapsulated with bioactive agents (**b**) Molecular structure of PEG.

Nanoliposomes are tiny, artificially created spherical vesicles primarily made from cholesterol and phospholipids. They display a size range of 30 nm to a few micrometers. These drug delivery systems display hydrophilic and hydrophobic features (Figure 3a). Numerous anticancer drug-loaded nanoliposomes have been fabricated for the treatment of breast and prostate cancer using PEGylation technology. PEG is a synthetic, highly hydrophilic, and versatile polymer with several functional end groups and diverse chain lengths [17]. It has broad-spectrum applications in biological, biochemical, and biophysical research and as an ingredient in cosmetic products, the food industry, and medical therapy [18]. The interesting properties of PEG that have attracted the attention of biomedical researchers include non-toxicity and non-immunogenic reactions and increasing drug stability. PEG molecules are chemically bonded to the formulations in a process called PEGylation. PEG has significantly increased the potency and pharmacodynamic activities of various drugs, such as interleukin -6, interferon a2a, and tumor necrosis factor [19,20].

PEGylation is explained as the covalent conjugation of PEG to nanocarriers, such as nanoliposomes, dendrimers, micelles, lipid nanoparticles, or biomolecules, such as therapeutic proteins [21]. PEGylating was essentially intended to enhance the pharmacokinetic properties of the PEGylated products and subsequently improve their therapeutic activity [21]. PEGylated nanoliposomes boost drug loading capacity by as much as 90%. PEGylated technology is a promising drug delivery system, as it effectively delivers hydrophobic and hydrophilic bioactive molecules [22,23]. PEG is an important part of many nanoparticles because it dissolves easily in water, helps control drug release profiles, and stays stable in the bloodstream. PEGylated nanoliposomes lowers the quantity of encapsulated chemicals that leak out. Henceforth, PEGylated nanoliposomes not only enhance the bioavailability of the encapsulated drug but also provide excellent targeting of cancer cells [20]. PEGylated nanoliposomes significantly enhance the ability of drugs to penetrate cancer cells. This enhancement occurs through the phenomenon known as the enhanced permeability and retention (EPR) effect, which is particularly evident in vascular systems of tumours that have leaky blood vessels. As a result, drugs modified with PEG can effectively accumulate within these cancerous tissues. Furthermore, PEGylation also extends the drug’s plasma half-life. This dual action of improving cellular uptake and prolonging circulation time enhances the therapeutic efficacy of the drug in cancer treatment [24,25]. The schematic presentation of nanoliposomes loaded with bioactive agents is shown in Figure 3b below.

PEGs are Generally Recognized as safe (GRAS) due to their excellent biocompatibility and they have been approved by FDA for biological and medical applications. PEGs of molecular weights of 1000–5000 Da are reported to be safe for administration in 10% solutions to rats, monkeys, and guinea pigs [21]. Moreover, developing the “stealth” properties of PEGylated nanoliposomes is a vital aspect of drug delivery, as the efficiency of nanocarriers is closely associated with their circulation time. To acquire “stealth” nanoliposomes, alteration of the bilayer surface with inert polymers, which control semi-phase processes and avoid interaction of nanoliposomes and blood components, displaying the so-called “stealth” features, is employed. Prolonged circulation time, low aggregation in blood serum and storage stability, and low mononuclear phagocyte system (MPS) cellular uptake are major benefits of “stealth’’ PEGylated nanoliposomes [26]. Two methods of PEGylation are explored in nanoliposomal formulations: pre-insertion method (mixing of PEG-lipids to lipid composition before formation of nanoliposome) or post-insertion method (addition of PEG-lipids and nanoliposomal dispersion) [27].

Although PEGylated nanoliposomes have excellent therapeutic outcomes in cancer, some limitations of PEG must be considered during their formulation. Disadvantages of PEG are as follows: PEG in solid-state or solution can undergo significant degradation under heat, making it unstable and unsafe in pharmaceutical applications [21], slow degradation of PEG only by cytochrome P-450, alcoholic dehydrogenase, and aldehyde dehydrogenase [28], and toxicity of PEG polymerization residues (e.g., formaldehyde and ethylene oxide) [29]. Moreover, PEG can induce immunogenicity and antigenicity which are caused by the production of anti-PEG antibodies. These anti-PEG antibodies can stimulate hypersensitivity reactions (HSRs). The development of HSRs toward the PEGylated nanocarriers is still a problem concerning their medical applications [30]. PEGylated nanocarriers can prime the host immune system by complement system activation stimulating acute HSRs [30]. These limitations of PEGs can be solved by using an innovative drug delivery approach or the judicious selection of PEG (for example, branched PEG is found to be less immunogenic) [17]. Another strategy is administering short oligomers of PEG before the PEGylated drug to prevent the development of immune complexes [17].

Betker and Gomez et al. discovered that PEGylated liposomes exhibited greater protein adsorption on the nanoparticle surface in comparison to conventional liposomes, which raised questions about the non-immunogenic characteristics of PEG [31]. However, in an animal study involving rabbits, Sherman et al. [32] investigated how the methoxy group (-OCH_3_) affects immune responses to proteins that are conjugated with mPEG. They emphasized the possible advantages of replacing mPEG with hydroxyl PEG (HO-PEG). Recently, their study in mice examined how the terminal groups of PEG influence the production of anti-PEG IgM (immunogenicity), the cross-reactivity of the generated anti-PEG IgM (antigenicity), and the systemic elimination of second doses of modified liposomes. They incorporated different functional groups, including amino (NH_2_), methoxy (OCH_3_), carboxyl (COOH), and hydroxyl (OH), at the terminal ends of PEG to study their impact on the induction of anti-PEG IgM. The findings indicated that liposomes altered with HO-PEG resulted in minimal levels of anti-PEG IgM after a single intravenous administration. Repeated injections of HO-PEG liposomes revealed that an initial dose led to the accelerated clearance of a subsequent dose. In vitro experiments demonstrated that HO-PEG liposomes activated the complement system in the presence of anti-PEG IgM. This indicates that the anti-PEG IgM, which is produced following the first dose, triggers the rapid elimination of the second dose through complement activation [33]. The use of empty liposomes (Doxebo) before administering pegylated drugs has been proposed as a strategy to reduce infusion reactions. However, it remains to be confirmed whether sufficient doses can be used to prevent the development of anti-PEG antibody responses [34]. PEGylation has enhanced stability issues. It is currently considered an effective approach to increasing stability and prolonging liposomes’ in vivo circulation time [35].

## 3. PEGylated Nanoliposomes in Breast and Prostate Cancer Therapy

### 3.1. PEGylated Nanoliposomes for Breast Cancer Therapy

**Figure 4:**
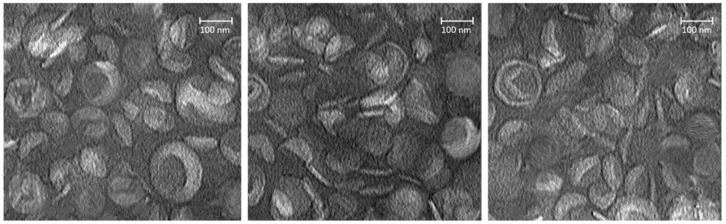
TEM micrographs of negatively stained nanoliposomal formulations. Reproduced with copyright permission from Elsevier [30].

There are several preclinical studies that have shown anticancer drug-encapsulated PEGylated nanoliposomes as potential nanotherapeutics for breast cancer therapy (Table 1). Mirzavi et al. formulated PEGylated nanoliposomes encapsulated with combretastatin A4 (CA4) from hydrogenated soy phosphatidylcholine (HSPC), 1,2-distearoyl-sn-glycero-3-phosphoethanolamine-N-[methoxy (polyethylene glycol)_2000_] (DSPE-mPEG_2000_) and cholesterol for breast cancer treatment [36]. The transmission electron microscopy (TEM) (Figure 4) and dynamic light scattering (DLS) analysis of HSPC/DSPE-mPEG_2000_/Cholesterol/CA4 nanoliposomes displayed an average particle size of 100 to 150 nm with negative surface charge and PDI < 0.3, demonstrating that these PEGylated nanoliposomes sizes were homogeneously distributed. In vitro, drug release tests at acidic conditions (pH 5.5 and 5.5, mimicking tumor microenvironment) and physiological conditions (pH 7.4, 37 °C) exhibited a pH-responsive CA4 release manner from nanoliposomes because higher levels of CA4 were released at pH 5.5 and pH 6.5 than at pH 7.4, indicating that CA4-encapsulated nanoliposomes can lead to superior cytotoxicity against cancer than in normal cells. The in vivo evaluations using the 4T1 xenograft BALB/c mouse model showed that CA4-encapsulated nanoliposomes started proliferating at the tumor site 3 h after injection, with the most significant tumor accumulation at 24 h. These in vivo studies further demonstrated that HSPC/DSPE-mPEG_2000_/Cholesterol/CA4 nanoliposomes significantly reduce tumor growth when compared to free CA4 without significant weight loss in the mice, revealing excellent antitumor efficacy against breast cancer with no side effects [36].

**Figure 5:**
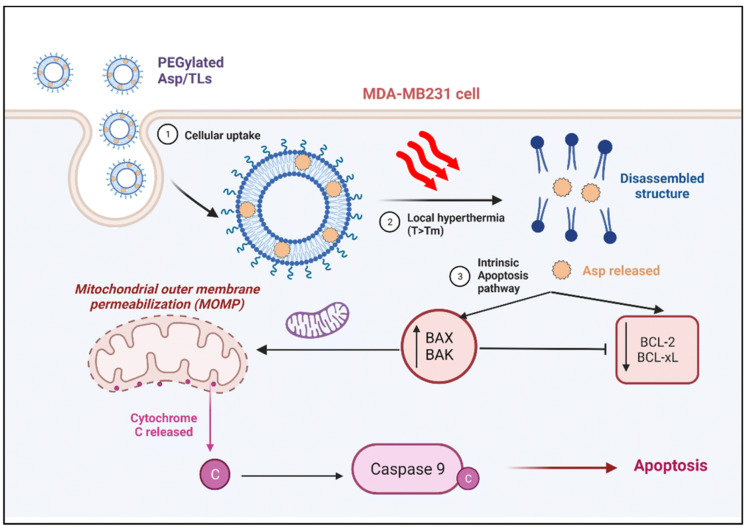
The molecular mechanism underlying the effectiveness of optimal Asp/TLs in triple-negative breast cancer focuses on their ability to induce apoptosis in the invasive MDA-MB-231 cell line. Reproduced with copyright permission from the Royal Society of Chemistry (RSC) [39].

The in vitro analysis of curcumin derivatives-encapsulated HSPC/DSPE-mPEG_2000_/Cholesterol nanoliposomes reported by Hatamipour et al. displayed higher cytotoxic effects against breast cancer 4T1 cells than in the human NIH 3T3 cell line, showing superior anticancer activity against breast cancer with reduced side effects on normal cells [37]. Celia et al. formulated DPPC (1,2-dipalmitoyl-sn-glycero-3-phosphocholinemonohydrate)/Cholesterol/DSPE-mPEG_2000_ nanoliposomes co-encapsulated with gemcitabine and paclitaxel for metastatic breast cancer treatment. The pharmacokinetic studies of dual drug-loaded PEGylated nanoliposomes showed an increased half-life of 1.7-fold and 6.3-fold for paclitaxel and gemcitabine hydrochloride, respectively. In vivo anticancer experiments exhibited tumor growth in metastatic triple-negative breast cancer cell line (MDA-MB-231)-bearing nude mice was significantly inhibited in response to dual drug-loaded PEGylated nanoliposomes compared to plain PEGylated nanoliposomal formulation and combination therapy with Taxol and Gemzar, demonstrating remarkable antitumor effectiveness, resulting from the combined synergistic effects of paclitaxel and gemcitabine [38]. Sedky et al. prepared thermo-responsive DPPC/Cholesterol/DSPE-mPEG_2000_ nanoliposomes encapsulated with asplatin [platinum (IV) anticancer complex] for breast cancer treatment. These nanoliposomes displayed a round shape with the thermo-triggered release of asplatin at 38 °C, reaching the highest drug release at 40 °C. The in vitro cytotoxicity studies of asplatin-loaded nanoliposomes exhibited the highest cytotoxic efficacy upon exposure to mild hyperthermia (40 °C) against the MDA-MB-231 cells as contrasted to other formulations [39]. The mechanism of action of these asplatin-loaded nanoliposomes against breast cancer cells is displayed in Figure 5.

Al-Samydai et al. prepared nanoliposomes made of DPPC, cholesterol, and DSPE combined with PEG_2000_. These nanoliposomes were designed to deliver capsaicin for treating breast cancer. The TEM results exhibited that Capsaicin-loaded nanoliposomes are uniformly sized and spherically shaped with an average particle size that ranges between 99.48 and 123.68 nm. The in vitro cytotoxicity analysis showed that Capsaicin-loaded nanoliposomes had superior cytotoxic effects towards breast cancer cell lines (MCF7 and MDA-MB-231 cells) than the free Capsaicin, revealing the potential of PEGylated nanoliposomes to improve biological efficacy of anticancer drugs [40]. Behdarvand et al. formulated Tamoxifen-loaded PEGylated nanoliposomes from poly(D,L-lactide) (PLA) core and 1,2-dipalmitoyl-sn-glycero-3-phosphoethanolamine-N-[methoxy(polyethyleneglycol)-2000] (DPPE-PEG) for breast cancer treatment. In vitro drug release studies displayed the highest cumulative Tamoxifen release percentage at 40 °C and pH of 4, indicating that these nanoliposomes are pH- and temperature-responsive, and can contribute to tumor targeting. The tumor microenvironment exhibits acidity and elevated temperatures [41].

Nandi et al. fabricated sirolimus-encapsulated PEGylated nanoliposomes using distearoylphosphatidylcholine (DSPC) or DPPC and DSPE for breast cancer treatment. The particle size analysis of nanoliposomes showed a mean average particle size of 170–200 nm with a high drug encapsulation efficiency of 90–95%. The in vitro drug release profile of PEGylated nanoliposomes displayed initial burst drug release that was due to the presence of free sirolimus on the surface of nanoliposomes; this was followed by a sustained release attributed to the PEGylation of nanoliposomes. The MTT assay showed that sirolimus-loaded nanoliposomes reduced cell viability by 25% against BT-474 breast cancer cells after 24 h while plain nanoliposomes reduced cell viability by only 8.0% [42]. The in vivo studies of PEGylated HSPC/mPEG_2000_-DSPE/DSPG/Cholesterol nanoliposomes encapsulated with docetaxel using BALB/c mice bearing TUBO or 4T1 breast carcinoma tumors was by reported Vakili-Ghartavol et al. which showed a significantly greater delay in tumor growth and a longer survival time compared to the Taxotere group at the same dose of 8 mg/kg [43]. The polytherapy of Docetaxel and metformin using HSPC/DPPG/mPEG_2000_-DSPE/Cholesterol nanoliposomes reported by Vakili-Ghartavol et al. showed superior antitumor efficacy and increased life span of BALB/c mice bearing 4T1 breast carcinoma tumors [44].

Shahbazi et al. formulated letrozole-loaded Phosphatidylcholine/mPEG_20000_/cholesterol nanoliposomes with particle sizes that ranged between 159.7 and 316.4 nm. The cytotoxicity studies of letrozole-loaded PEGylated nanoliposomes at 200 μg/mL concentration induced a superior reduction in MCF-7 cancer cell viability with an IC_50_ value of 18.60 μg/mL than the free letrozole, plain nanoliposomes, and letrozole-encapsulated non-PEGylated nanoliposomes that possessed IC_50_ values 119.5, 2774 and 57.58 μg/mL after 48 h, respectively. Furthermore, the encapsulation of letrozole in the PEGylated nanoliposomes showed lower toxicity towards normal (HFF) cells as contrasted to the ones of the pristine letrozole. These results showed that PEGylation of nanoliposomes significantly improved the anticancer efficacy of letrozole with reduced toxic effects on normal cells [45].

Mohammadinezhad et al. prepared lecithin/cholesterol/PEG_3350_ nanoliposomes loaded with cisplatin for breast cancer therapy. The anticancer studies showed that drug-encapsulated PEGylated nanoliposomes resulted in higher cytotoxic effects against breast cancer lines with an IC_50_ value of 58.1 ± 22.7 μg/mL when compared to free cisplatin (140.8 ± 51.8 μg/mL), suggesting excellent anticancer efficacy of cisplatin-encapsulated nanoliposomes [46]. Zarei and Yaraghtala formulated gingerol-loaded PEGylated phosphatidylcholine/cholesterol nanoliposomes that exhibited spherical vesicles and a size range of 111–184.5 nm with a good loading efficiency of 96.5%. The in vitro cytotoxicity of gingerol-loaded PEGylated nanoliposomes utilizing MTT assay showed superior cytotoxic effects on breast cancer cell lines (MCF-7) with IC_50_ value of 60.5 µM when compared to pristine gingerol (IC_50_ value of 91.35 µM), suggesting good anticancer activity than free drug [47]. The dual drug-loaded palmitoyl-X-MMP9/mPEG_2000_ nanoliposomes co-encapsulated with Erlotinib and Doxorubicin reported by Kim et al. showed higher cellular uptake and cytotoxic effects against MDA-MB-231 cells, which was due to synergistic anticancer effects [48].

Zhang et al. formulated DPPC/DSPE-mPEG_2000_/cholesterol nanoliposomes loaded with melanin for photothermal breast cancer therapy. The anticancer experiments of melanin-loaded nanoliposomes, in vivo, revealed desirable photothermal-conversion efficiency that accomplished complete elimination of tumors in MDA-MB-231 cancer-bearing mice, displaying notable photothermal-based therapeutic efficacy [49]. The DPPC/DSPE-mPEG_2000_/cholesterol nanoliposomes co-loaded with indocyanine green and perfluorooctyl bromide reported by Sheng et al. significantly induced up to approximately 93% cell death of MDA-MB-231 cells in vitro (CCK-8 assay), revealing excellent anticancer activity [50]. Shahbazian et al. fabricated PEG 3350/Cholesterol nanoliposomes encapsulated with trans-anethole that exhibited slowly sustained release (7% in 38 h), which was due to the presence of PEG. The in vitro anticancer using MTT assay showed that the trans-anethole-loaded nanoliposomes displayed high cytotoxicity against MCF-7 and T47D cell lines, which were determined to be nine and eight-fold higher than the standard drugs, respectively [51].

Zhang et al. fabricated DSPC/DOPE/PEG_2000_ nanoliposomes loaded with C6-ceramide for cancer therapy. The anticancer studies of C6-ceramide-loaded PEGylated nanoliposomes significantly induced apoptosis of MDA-MB-231 cells overtime [52]. The trastuzumab-encapsulated DPPC/DPPG/cholesterol/PEG_2000_ nanoliposomes reported by Golkar et al. exhibited high cytotoxic effects against MCF-7 cells that were dependent on the content of trastuzumab [53]. Chen et al. prepared HSPC/DOPE/mPEG_2000_ nanoliposomes co-encapsulated with imatinib and doxorubicin for breast cancer therapy. The DLS analysis of nanoliposomes showed particle size, negative surface charge, and PDI of 159 ± 6 nm, −20 ± 2 mV, and 0.103 ± 0.006. The in vitro drug release studies showed that both imatinib and doxorubicin release kinetics were pH-dependent, whereby the initial release at pH 5.5 was slightly higher than at pH 6.5, indicating suitable release mechanism in the acidic microenvironment of cancer. The anticancer analysis in nude mice bearing MCF-7/ADR xenograft tumors showed superior antitumor effects for dual drug-loaded PEGylated nanoliposomes than free drugs [54].

Sauvage et al. formulated PC/cholesterol/DSPE-PEG_2000_ nanoliposomes loaded with novobiocin analogue, 6BrCaQ for breast cancer therapy. The in vivo studies of drug-loaded PEGylated nanoliposomes in MDA-MB-231 tumor-bearing female mice displayed significantly reduced tumor volume than the free drug [55]. Talazoparib-loaded DPPC/1,2-dioleoyl-3-trimethyl-ammonium propane/(DOTAP)/cholesterol/DSPE-PEG_2000_ nanoliposomes reported by Zhang et al. showed enhanced anticancer activity and reduces off-target toxicities in BRCA-deficient breast cancer mice [56]. Farzad et al. fabricated Maleimide/PEG_2000_/DSPE nanoliposomes loaded with P435 HER2/neu-derived peptide that exhibited lower tumor size and a longer survival time in TUBO mice model, suggesting superior antitumor efficacy with good biocompatibility [57]. The P5 (HER2)/neu-derived peptide-loaded Maleimide/PEG_2000_/DSPE nanoliposomes reported by Rastakhiz et al. demonstrated superior antitumor effects in BALB/c mice bearing TUBO carcinoma [58]. Moreover, Razazan et al. reported HER2/neu-derived peptide-encapsulated maleimide/DOPE/cholesterol/mPEG_2000_-DSPE nanoliposomes that showed good anticancer activity in TUBO mice model [59].

Zarei and Yaraghtala prepared PC/cholesterol/PEG_2000_ nanoliposomes encapsulated with gingerol for the treatment of breast cancer. The loading efficiency of PEGylated and non-pegylated nanoliposomes was 92.2% and 96.5%, respectively, showing that PEGylation significantly improved loading efficiency. The cytotoxicity studies using MTT assay displayed anticancer efficacy of gingerol-loaded PEGylated nanoliposomes was superior to that of gingerol-loaded non-PEGylated nanoliposomes against MCF-7 cells. Nevertheless, both liposomal formulations were more cytotoxic than the free drug [47]. Chiani et al. formulated folic acid conjugated DSPC/cholesterol/mPEG_2000_-DSPE nanoliposomes encapsulated with bleomycin for breast cancer treatment. The DLS analysis of nanoliposomes showed a mean particle size, zeta potential, and narrow PDI of 100 nm, −36 mV, and 0.25, respectively. The in vitro cytotoxicity assay showed that these nanoliposomes had higher cytotoxic effects than pristine bleomycin against MCF-7cells, indicating improved anticancer activity of bleomycin encapsulated into PEGylated nanoliposomes [60]. The doxorubicin-loaded DPPC/cholesterol/DSPE-PEG_2000_ nanoliposomes reported by Shi et al. displayed superior cytotoxic effects against MCF-7/MDR cells than free drug, indicating significant anticancer efficacy [61].

### 3.2. PEGylated Nanoliposomes for Prostate Cancer Therapy

**Figure 6:**
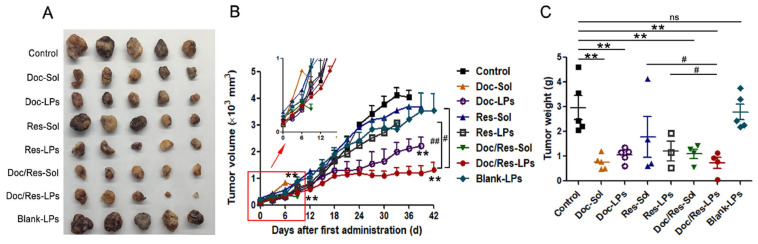
(**A**) Photographs of tumors, (**B**) tumor volume changes, (**C**) tumor weight changes on PC3 bearing male Balb/c nude mice after intravenous injection with different formulations. *p* < 0.05, ** *p* < 0.01, a statistically significant difference compared with the control group; # *p* < 0.05, ## *p* < 0.01, a statistically significant difference contrasted with Doc/Res-LPs group. ns; not significant. Data were given as mean ± SD (*n* = 5). Reproduced with copyright permission from Elsevier [62].

Some biomedical researchers reported using PEGylated nanoliposomes that contain anticancer drugs to treat prostate cancer (Table 2). Zhang et al. fabricated soy lecithin/cholesterol/DSPE-MPEG_2000_ nanoliposomes co-encapsulated with docetaxel and resveratrol for prostate cancer therapy [62]. The particle size evaluation of dual drug-loaded nanoliposomes displayed an average diameter of 99.67 nm with a sphere-like shape. The in vitro drug release experiments of PEGylated nanoliposomes at physiological conditions exhibited sustained release of docetaxel and resveratrol, revealing their potential to reduce drug toxicity on normal cells. The in vivo experiments of dual drug-loaded nanoliposomes utilizing PC3-bearing mice showed a significant reduction of tumor volume/weight compared to the saline-treated groups. However, it was superior to the group treated with docetaxel or docetaxel/resveratrol solution (Figure 6) [62]. Ma et al. prepared egg phosphatidylcholine (EPC)/cholesterol/DSPE-PEG_2000_ nanoliposomes loaded with curcumin for prostate cancer therapy [63]. The in vitro drug release experiments of curcumin-encapsulated nanoliposomes at physiological conditions showed initial rapid drug release behavior within (20–25%) followed by a sustained drug release pattern to approximately 68%. The studies on cellular uptake of curcumin-encapsulated nanoliposomes into prostate cancer cells using DU145 was more effective than the free curcumin. This enhanced internalization could lead to improved anticancer efficacy. The in vivo studies employing DU145 prostate carcinoma-bearing mice demonstrated that curcumin-encapsulated nanoliposomes significantly reduced tumor growth more than free drugs, indicating excellent antitumor activity against prostate cancer [63].

Tian et al. formulated PSMA-conjugated PEGylated L-α-phosphatidylcholine/cholesterol/DSPE-PEG_2000_ nanoliposomes co-encapsulated with plumbagin and genistein for prostate cancer therapy [64]. These nanoliposomes showed an excellent encapsulation efficiency of 72.20% and 87.90% of genistein and plumbagin, respectively, demonstrating that genistein and plumbagin did not interfere during co-encapsulation. The in vitro experiments displayed slow and sustained release of plumbagin and genistein from nanoliposomes that can lead to improved anticancer efficacy. The MTT assay showed more than 90% cell death against PC-3 and LNCaP prostate cancer cells when incubated with dual drug-loaded nanoliposomes, indicating superior anticancer efficacy. Moreover, Tian et al. developed EPC/DPPE-PEG_2000_ nanoliposomes for the co-delivery of celecoxib and genistein, aiming to enhance therapeutic efficacy in the treatment of prostate cancer. The TEM analysis showed spherical structures with a mean particle size distribution between 85 and 110 nm. The MTT assay showed that the dual drug-encapsulated nanoliposomes significantly lowered cell viability of prostate (PC3) cancer cells without causing any cell death of fibroblast cells, revealing superior anticancer efficacy with good cytocompatibility and non-toxicity on normal cells [64]. The L-α-phosphatidylcholine/DPPE-PEG-2000 nanoliposomes co-loaded with celecoxib and genistein reported by Tian et al. displayed significantly inhibited the proliferation of prostate cancer (PC-3) cells up to 90% without any toxicity towards normal fibroblast cells, indicating superior anticancer activity with excellent cytocompatibility [65].

The L-α-phosphatidylcholine/cholesterol/DSPE-PEG_2000_ nanoliposomes co-encapsulated with genistein and plumbagin reported by Song et al. significantly induced a reduction in tumor growth of mice bearing PC3 and LNCaP cells in mice bearing PC3 and LNCaP prostate cancer cells than the free drugs. These findings revealed that co-encapsulation of anticancer drugs in PEGylated significantly resulted in synergistic antitumor effects [66]. Nassira et al. fabricated oleuropein-encapsulated folate-modified DSPE-mPEG_2000_/cholesterol nanoliposomes for prostate cancer therapy. The in vitro cytotoxicity tests using MTT assay exhibited the IC_50_ values of 132.23 ± 1.12 μM and 391.47 ± 1.22 μM for oleuropein-encapsulated nanoliposomes and free oleuropein when incubated with human prostate cancer (22Rv1) cells, respectively. These therapeutic outcomes demonstrated that the PEGylated nanoliposomes improved the anticancer effects of oleuropein [67]. Shao et al. formulated 1,2-dioleoyl-sn-glycero-3-phosphatidylcholine (DOPC)/DSPE/PEG nanoliposomes co-encapsulated with TE small interfering RNA (siRNA) and docetaxel for prostate cancer therapy. In vivo experiments of these nanoliposomes using VCaP xenograft mice models showed reduced tumor growth of more than 84%, indicating excellent antitumor efficacy against prostate cancer [68].

## 4. Nanoliposomes in Clinical Trials for Cancer Therapy

There are various clinical trials that have shown the potential of nanoliposomes in cancer therapy (Table 3). Taléns-Visconti et al. documented a clinical trial using nanoliposomes known as Liposomal SN-38 to treat metastatic colorectal cancer. The results revealed that this nanoliposome demonstrated promising progress in reducing toxicity, increasing survival rates, and achieving a positive response [69]. Muthu et al. mentioned the encapsulation of liposomal cisplatin within liposomes, which had an average diameter of 110 nm. Lipoplatin has significantly lowered kidney damage, nerve damage, ear toxicity, bone marrow toxicity, vomiting, as well as the feelings of nausea and weakness caused by cisplatin in Phase I, II, and III clinical trials while maintaining or enhancing its effectiveness compared to cisplatin [70].

According to He et al., SN38 conjugated with lysophospholipid utilizing a cleavable disulfide bond linker was developed. The conjugate was used to form liposomes using the thin film method. The results indicated a narrow size distribution of 172.8 ± 10.5 nm and a negatively charged zeta potential (−8.9 ± 0.3 mV). Testing for cytotoxicity in vitro towards MCF-7 and A549 cells exhibited enhanced effectiveness in combating cancer. The researchers concluded that the nanoliposome might be an effective delivery system for SN38 in cancer therapy due to its redox responsiveness [71]. Hadisadegh et al. created nanoscale liposomes containing carboplatin using reverse-phase evaporation. The impact of these nanoliposomes on MDA-MB 231 breast cancer cells was researched. The findings demonstrated that the liposomal nanoparticles had a negative zeta potential of −18.2 mV and an average size of 278 nm. The drug loading capacity was 2.2%, and drug encapsulation efficiency was 58.5%. It was noted that the nanoliposomal form of carboplatin was more effective toward the MDA-MB 231 breast cancer cell line than the free drug [72]. Adekiya et al. reported the combination of nanoliposomal irinotecan with fluorouracil and folinic acid. Combining 5-FU/folinic acid with nanoliposomal irinotecan is a significant advancement in enhancing treatment choices for patients with advanced pancreatic cancer who have advanced to the second line. Moreover, the innovative drug formulation provides pharmacokinetic benefits that provide a foundation for additional clinical trials in diverse pancreatic cancer scenarios and other types of cancer [73]. Zhang et al. prepared an elastin-like polypeptide/liposomal-encapsulated docetaxel at high concentrations. This innovative drug delivery system effectively reduced the viability of prostate cancer cells in a lab setting by delivering docetaxel specifically to the cells [74].

Khosravani et al. used thin-layer lipid hydration techniques to synthesize liposomal co-loaded curcumin and arsenic trioxide, which were amended by RGD peptide. This was done for the purpose of treating prostate cancer. The liposomal co-loaded curcumin and arsenic trioxide improved by Arginylglycylaspartic acid peptide had a particle size of around 100 nm, and the encapsulation efficiency was 99.52% for arsenic trioxide and 70.61% for Curcumin. The nanoliposome demonstrated an improved anti-proliferative effect, increasing the percentage of apoptotic cells to 98 ± 1.85% (*p* < 0.0001) and reducing the EGFR gene expression level (*p* < 0.001) in the tested cell line. The findings suggest that our co-delivery system of Liposomal co-loaded curcumin and arsenic trioxide improved by Arginylglycylaspartic acid peptide holds promise for prostate cancer therapy [75].

The researchers Nezir et al. formulated nanoliposomes using Poly (2-ethyl-2-oxazoline) dioleoyl phosphatidylethanolamine. These nanoliposomes were attached to a peptide that targets prostate-specific membrane antigens and were filled with BikDDA, a modified version of the proapoptotic protein Bik. The findings of BikDDA expression increased in 22Rv1 cells, leading to cell death. Moreover, CD-1 nude mice xenografts treated with the compound exhibited notable tumor regression. The suggestion indicated the results proposed that Poly (2-ethyl-2-oxazoline) dioleoyl phosphatidylethanolamine-BikDDA nanoliposomes can function as effective gene transporters for combating prostate cancer [76]. A different team of scientists studied the application of liposomes containing curcumin by sonication at an average size of 100–150 nm and covered with antibodies that target the prostate membrane antigen. This method aims to enhance the effectiveness of curcumin as a therapeutic drug. The findings showed a decrease in the occurrence of prostate cancer [77].

## 5. Conclusions and Future Perspective

PEGylation technology has been shown to be a promising approach that can be used for the formulation of nanoliposomes. Several biomedical researchers have reported that PEGylated nanoliposomes loaded with bioactive agents are effective systems for treating breast and prostate cancer. Most of these nanoliposomes displayed particle sizes that are approximately 100 nm in diameter, which are considered suitable sizes to be easily internalized by cancer cells. The other properties that make PEGylated nanoliposomes potential candidates in cancer treatment include a controlled and sustained drug release profile, enhanced cancer targeting, reduced drug toxicity towards normal cells, good biocompatibility, etc. The co-encapsulation of anticancer drugs into the nanoliposomes significantly resulted in synergistic anticancer effects to decrease the proliferation of breast and prostate cancer cells. Henceforth, additional investigations into the optimization of PEGylated nanoliposomes, their mode of action, and translation into clinical applications are very crucial to understanding their full therapeutic effectiveness and enhancing therapeutic outcomes for breast and prostate cancer patients. The PEGylated nanoliposomes were more effective against breast cancer cell lines than prostate cancer cells, which might be the reason why their research trends have been mainly focused on breast cancer than prostate cancer.

## Figures and Tables

**Table 1:** PEGylated Nanoliposomes for Breast Cancer Therapy.

Composition of PEGylated Nanoliposomes	Encapsulated Drugs	Particle Size (nm)	Main Findings	Reference
HSPC, DSPE, mPEG_2000_, and cholesterol	Combretastatin A4	100–150	pH-responsive drug release behavior and excellent anticancer effectiveness	[36]
HSPC, DSPE, mPEG_2000_, and cholesterol	Curcumin derivatives	104.4–119.4	Higher cytotoxic effects against breast cancer 4T1 cells as contrasted to human NIH 3T3 cell line	[37]
DPPC, DSPE, mPEG_2000_, and cholesterol	Gemcitabine and paclitaxel	150	Prolonged drug half-life and synergistic anticancer effects	[38]
DPPC, DSPE, mPEG_2000_, and cholesterol	Asplitin	114.05	Thermo-triggered release of asplatin with good anticancer activity	[39]
DPPC, Cholesterol, DSPE, and PEG_2000_	Capsaicin	99.48–123.68	Superior anticancer effects	[40]
PLA, DPPE, and PEG_2000_	Tamoxifen	57–159	Controlled drug release mechanism	[41]
DSPC or DPPC, DSPE and PEG_2000_	Sirolimus	170–200	Sustained drug release profile and improved anticancer activity	[42]
HSPC, mPEG_2000_-DSPE, DSPG, and Cholesterol	Docetaxel	115	Delayed tumor growth and prolonged survival time	[43]
HSPC, DPPG, mPEG_2000_-DSPE, and Cholesterol	Docetaxel and metformin	99.98–160.8	Superior antitumor activity and prolonged life span of treated mice	[44]
Phosphatidylcholine, mPEG_20000_, and cholesterol	Letrozole	159.7–316.4	Excellent anticancer efficacy and cytocompatibility on normal cells	[45]
Lecithin, cholesterol, and PEG3350	Cisplatin	342.3	Higher cytotoxic effects against breast cancer lines	[46]
Phosphatidylcholine, cholesterol, and mPEG_2000_	Gingerol	111–184.5	Superior anticancer activity when compared with free drug	[47]
Palmitoyl-X-MMP9 and mPEG_2000_	Erlotinib and Doxorubicin	68.3–109.2	High cellular uptake and good anticancer effects	[48]
DPPC, DSPE-mPEG_2000_ and cholesterol	Melanin	299.3–300.5	Full elimination of tumors	[49]
DPPC, DSPE-mPEG_2000_, and cholesterol	Indocyanine green and perfluorooctyl bromide	-	Excellent anticancer activity	[50]
PEG 3350 and Cholesterol	Trans-anethole	37	Initial burst drug release and good anticancer efficacy	[51]
DSPC, DOPE, and PEG_2000_	C6-ceramide	-	Induced apoptosis	[52]
DPPC, DPPG, cholesterol, and PEG_2000_	Trastuzumab	53–76	High cytotoxic effects against cancer cells	[53]
HSPC, DOPE, and mPEG_2000_	Imatinib and doxorubicin	159	pH-dependent drug release and superior antitumor effects	[54]
PC, cholesterol, and DSPE-PEG_2000_	Novobiocin analogue 6BrCaQ	124	Reduced tumor volume	[55]
DPPC, DOTAP, cholesterol, and DSPE-PEG_2000_	Talazoparib	4.5	Enhanced anticancer activity and reduces off-target toxicities	[56]
Maleimide, PEG_2000_, and DSPE	P435 HER2/neu-derived peptide	159.1–183.1	Reduced tumor volume and longer survival time	[57]
Maleimide, and PEG_2000_-DSPE	P5 (HER2)/neu-derived peptide	144.1–169	Superior antitumor effects	[58]
Maleimide, DOPE, cholesterol, and mPEG_2000_-DSPE	HER2/neu-derived peptide	120–160	Good anticancer activity	[59]
PC, cholesterol, and PEG_2000_	Gingerol	111–184.5	Superior anticancer activity	[47]
DSPC, cholesterol, and mPEG_2000_-DSPE	Bleomycin	100	Higher cytotoxic effects against cancer cells	[60]
DPPC, cholesterol, and DSPE-PEG_2000_	Doxorubicin	126.6	Higher cytotoxic effects against cancer cells	[61]

**Table 2:** PEGylated Nanoliposomes for Prostate Cancer Therapy.

Composition of PEGylated Nanoliposomes	Encapsulated Drugs	Particle Size	Main Findings	Reference
Soy lecithin, cholesterol, DSPE, and MPEG_2000_	Docetaxel and resveratrol	99.67 nm	Sustained drug release profile and synergistic anticancer effects	[62]
EPC, cholesterol, DSPE-PEG_2000_	Curcumin	86.6 nm	High cellular uptake and lowered tumor growth	[63]
L-α-phosphatidylcholine, cholesterol, and DSPE-PEG_2000_	Plumbagin and genistein	60 to 90 nm	Slow and sustained release of drugs with superior anticancer efficacy	[64]
L-α-phosphatidylcholine, DPPE-PEG-2000	Celecoxib and genistein	85 to 110 nm	superior anticancer activity with excellent cytocompatibility	[65]
L-α-phosphatidylcholine, cholesterol, and DSPE-PEG_2000_	Genistein and plumbagin	100 nm	Synergistic antitumor effects	[66]
DSPE-mPEG_2000_ and cholesterol	Oleuropein	184.2 nm	Enhanced anticancer effects	[67]
DOPC, and DSPE, and PEG	siRNA and docetaxel	-	Reduced tumor growth	[68]

**Table 3:** Nanoliposomes in Clinical Trials for Cancer Therapy.

Type of Cancer	Nanoliposomes	Phase	Outcomes	References
Metastatic colorectal cancer	Liposomal-7-Ethyl-10-hydroxycamptothecin	II	Toxicity, objective response rate, and Progression-free survival	[69]
Multiple myeloma and Kaposi’s sarcoma	Lipoplatin^®^	I, II, and III	The renal toxicity, ototoxicity, myelotoxicity and peripheral neuropathy were decreased	[70]
MCF-7 and A549 cells	7-Ethyl-10-hydroxycamptothecin -loadedredox-sensitive liposomes	I	Suppress the evolvement of breast and lung cancer in living organisms by as much as 53.3%	[71]
MDA-MB 231 breast cancer cells	liposomes loaded with carboplatin	Late stage	The nanoliposomal form of carboplatin showed significantly increased potency against the MDA-MB 231 breast cancer cell in terms of cytotoxicity.	[72]
Pancreatic cancer	Irinotecan loaded liposome	Not specified	Enhancing alternative treatments for patients with advancing metastatic pancreatic cancer.	[73]
Prostate Cancer	elastin-like polypeptide/liposomal s	Not mentioned	Reduces the viability of prostate cancer cells	[74]
Prostate Cancer	RGD-decorated nanoliposomes arsenic trioxide and curcumin	Unmentioned	A co-delivery system showed great promise as a strategy	[75]
Prostate Cancer	MethodsPoly (2-ethyl-2-oxazoline)-dioleoyl phosphatidylethanolamine-nanoliposomes	Not stated	act as key gene carriers in the fight against prostate cancer.	[76]
Prostate Cancer	Liposome-curcumin and resveratrol	Not included	Decrease the occurrence of prostate cancer	[77]
